# Supplementary material for: Approved Ambiguities: An Analysis of Applications for the Ethical Review of Animal Research in Sweden—Focusing on Harm, Benefit, and the 3Rs
Source: Animals (Basel). 2025 Sep 23;15(19):2771. doi: 10.3390/ani15192771 (PMC12523415; doi:10.3390/ani15192771)
Supplement: Supplementary file 1 [file animals-15-02771-s001.zip › animals-3828846-supplementary.pdf]

Guide for Analysis of Applications for the Ethical Review of Animal Research

Analysis performed 2021 – 2022, documents from 2020

Grading:

In order to ascertain to what extent submitted applications contain the information specified in The Swedish Board of Agriculture's Regulations And General Advice on Laboratory Animals (SJVFS 2019:9, commonly known as and henceforth referred to as the L150), the information in question has been converted into "topics" each assigned criteria corresponding to different degrees of detail. Depending on the details provided, each topic will warrant a certain "grade".

Depending on how extensive different information *could* (and ideally *should*) be, e.g. if it could contain a justification or not, the available grades will be different for different topics. Most topics may be described by the researcher corresponding to the three grades: Y, I or N (see below). However, information regarding benefit provided by the researcher (in both the "main technical body" of the application form and the Non-Technical Project Summary) may be described to very varying degrees and can therefore receive any of all five available grades: Y!, Y, y, I or N. Proposed severity classification, on the other hand, may only be graded as present or not present: Y or N.

The criteria for different topics have to the greatest possible extent been formulated so as to contain the same wording as one another. However, individual nuances and specific requirements are present for some, and each topic must therefore be carefully analysed according to its specific requirements below. The listed topics are to be analysed and graded one by one.

<u>The standard grades are as follows:</u>

Y!, Y, y: Yes, information provided to varying degrees (see clarifications within each specific topic). Y! and y are not applicable grades for all topics.
I: Incomplete/Insufficient/Indeterminable information provided. This grade is not applicable for all topics.
N: No, information not provided.

<u>Additional grades:</u>

N/A: Information not applicable for a certain criterion.
Euth: Only applicable for how the animals are kept and cared for *after* the animal experiment to illustrate when all animals in a study will be euthanised at the end of a study leaving no animals alive after the experiment to be housed and cared for.

General aspects to consider:

To distinguish between the different grades or between *which requirements* have been fulfilled, focus should been put on *what precisely is asked of the applicant*. For example: a reply which answers the question *if* there are negative consequences for the animals is not the same as *which* negative consequences are predicted or *how* these are expected to affect the animals. Such nuances are vital for correct data analysis and must not be overlooked.

Please note: This guide is <u>not</u> intended for use to determine *if* an application should have been granted ethical approval or not. It is simply a tool for mapping which information has been provided by the researcher in the application form. This is then used to analyse whether or not the information lives up to the demands set by Directive 2010/63/EU implemented in the L150. The regulatory requirements have been determined through legal scholarship by use of a legal analytical approach*. All grades have in turn been constructed based on this information.

Translations of national regulations:

The English translations of the L150 below are derived from the translation by the Swedish Board of Agriculture used for education and training purposes, commissioned by the Nordic Consortium for Laboratory Animal Science Education and Training NCLASET (last updated 20-01-2020). The English translations of The Swedish Animal Welfare Act (SFS 2018:1192) has been obtained from the translations of the legislation found at the Swedish Government's homepage: https://www.government.se/information-material/2020/03/animal-welfare-act-20181192/.

Abbreviations:
AEC – Animal ethics committee
NTS – Non-technical project summary
3R – The principle of replacement, reduction & refinement of animal research

Important definitions:
The principle of the 3Rs is defined throughout this guide as in Chapter 1 Section 8 of the current L150 (SJVFS 2019:9):
"The principle to replace animal experiments with methods free of animals, to reduce the number of animals used in animal experiments and refine the use of animals in animal experiments to reduce their suffering or increase their wellbeing."
Harm-Benefit Analysis is defined throughout this guide as in Chapter 7 Section 13 of the current L150 (SJVFS 2019:9):
"During the review, the committee shall perform a harm, benefit analysis of the experiment from an ethical perspective, where the laboratory animal's suffering shall be weighed against the expected benefit that the animal experiment may result in for humans, animals, or the environment."

* described in the Methods section of the scientific paper for which this guide is included as supplementary material.

Guidelines for assessing the APPLICATIONS

Information within the "main technical body" of the application:

SJVFS 2019:9 Ch. 2 Section 15 The electronic application form for ethical approval shall contain relevant, and where applicable, motivated information about…

Purpose, benefit and proposed severity classification

SJVFS 2019:9 Ch. 2 Section 15 p. 4 The purpose of the experiment, its benefit, and proposed severity classification.

Please note:

- Purpose, benefit and proposed severity classification are analysed and graded separately.
- Purpose applies to the section of the application form where the applicant is asked to describe the purpose of their study in their own words. It does not correspond to the categories available as tick boxes in the digital application form where the applicant may for example tick "basic research", "research aimed at improving animal welfare" etc.
- Benefit within the scope of this legal requirement is not to be confused with benefit within the application form's request for an ethical weighing by the applicant (see below).

Purpose

Y The purpose of this particular project is described.

I The purpose of this particular project is not described. The purpose of similar research or of research within the same field might however be.
Generic phrases such as *"the study of immunological disorders in mice aims to give insight into the hereditary aspects of similar diseases in humans"* or *"a previous study aimed to…"* may be used.

N No mention of purpose.

Benefit

Y! The expected benefit of this particular project is described.
That there is expected benefit is not enough, rather *what* and/or *how great* said benefit is expected to be must be specified.
The *likelihood* of achieving said benefit(s) is also mentioned.

Y The expected benefit of this particular project is described.
That there is expected benefit is not enough, rather *what* and/or *how great* said

benefit is expected to be must be specified.
The *likelihood* of achieving said benefit(s) is not mentioned.

y	*That* there is expected benefit of this particular project is mentioned but not *what/how great* said benefit is expected to be.<br>The *likelihood* of achieving benefit(s) may or may not be mentioned.
I	The expected benefit of this particular project is not described but benefit(s) of similar research or of an enveloping broader research area is.<br>Generic phrases such as *"increased knowledge about the underlying causes of diseases is of great importance for the development of efficient drugs*" or "*the need for treatment is grea*t" may be used.
N	No mention of benefit.

Proposed severity classification

Y	A proposed severity classification is provided.
N	No mention of a proposed severity classification.

The 3Rs by the applicant

> SJVFS 2019:9 Ch. 2 Section 15 p. 6 *The need to use animals in the experiment. The number of laboratory animals and their origin as well as their developmental stage shall also be specified here.*
>
> SJVFS 2019:9 Ch. 2 Section 15 p. 7 *Use of methods for 3R in connection with using laboratory animals in the planned animal experiment.*
>
> SJVFS 2019:9 Ch. 2 Section 15 p. 9 *Strategy for obtaining qualified results and statistics with the lowest number of laboratory animals, with the lowest possible level of suffering for the laboratory animals and, where appropriate, environmental impact.*
>
> SJVFS 2019:9 Ch. 2 Section 15 p. 10 *Planned use of pain relief, anesthesia, and other methods to reduce the suffering of laboratory animals throughout their whole lifetime.*

Please note:

- The 3Rs are analysed and graded separately.
- The applicant is here according to the L150 requested to describe their *"methods"*, *"strategies"* and *"planned use of pain relief, anaesthesia and other methods"* to fulfil the 3Rs. Hence, similarly to how the 3R section of the non-technical project summary (NTS) is analysed (see below) it is not sufficient to simply say *that* a certain R has been considered. *How* this has been done should also be described for each.
- Due to the formulations of the content requirements in Article 37 and Annex VI of the Directive and Chapter 2 Section 15 p. 6 of the L150, the origin, estimated numbers, species and developmental stages of the animals used, including "relevance and justification", are listed below together with the 3Rs. In the application form, this information is

however not requested in direct relation to each other and as such, these criteria will be graded separately and may be fulfilled regardless of where throughout the application (including the NTPS) said information has been provided.

- Age, size, weight or other relative measures may all be acceptable to refer to developmental stage.

Replace

Y *How* Replacement has been considered for this particular project is described. *Why* animals need to be used and/or a certain animal model has been applied in this case is described and motivated (*relevance* and *justification* is given), not just that "*animal models are the only available options*" or "*the proposed model is standard*" etc.

I *That* Replacement has been considered for this particular project is described but not motivated.
Generic phrases such as *"the proposed model is the most established model"*, *"no alternative methods exist"* or *"for the proposed study, live animals are needed"* may be used.

N No mention of consideration or application of Replacement.

Origin

Y Origin is provided for all animals within the study and motivated (*relevance* and *justification* is given).

I Origin is provided to some extent, such as for some but not all animals, and may or may not be motivated.

N No mention of origin for any of the animals within the study.

Developmental stage(s)

Y Developmental stages are provided for all animals within the study and motivated (*relevance* and *justification* is given).

I Developmental stages are provided to some extent, such as for some but not all animals, and may or may not be motivated.

N No mention of developmental stages for any of the animals within the study.

Reduce

Y *How* Reduction has been applied and fulfilled within this particular project is described.
How the applicant has ensured the use of the least possible total number of animals for the study is described, for example by providing power calculations to determine sample size, and motivated (*relevance* and *justification* is given).

I *That* Reduction has been applied to and fulfilled within this particular project is described but not motivated.
Generic phrases such as *"by carefully planning the project, the number of animals needed has been kept to a minimum"* or *"only as many animals as are required to obtain scientifically valid results will be used"* may be used.

N No mention of consideration or application of Reduction.

Estimated numbers

Y Estimated numbers of animals are provided for all animal species used and motivated (*relevance* and *justification* is given).

I Estimated number of animals are provided to some extent, such as for some but not all animals, and may or may not be motivated.

N No mention of estimated number of animals for any animal species.

Refine

Y *How* Refinement has been applied and fulfilled within this particular project is described for all procedures.
How the applicant has considered and/or included Refinement measures is described and motivated (*relevance* and *justification* is given). This may be done separately for all animals/animal groups and/or procedures or may be described generally for all animals/animal groups and/or procedures, so long as it is clear that the refinement(s) apply to all animals and/or aspects of the study.

I *That* and/or *how* Refinement has been applied is described to some extent and may or may not be motivated. For example, it is described separately for some but not all animals/animal groups and/or procedures, or it is described generally for all animals/animal groups and/or procedures, but it is unclear if the refinement(s) apply to all animals and/or aspects of the study.
Generic phrases such as *"only educated staff handles the animals"* or *"the methods used are well-known and reliable"* may be used.

N No mention of consideration or application of Refinement.

Species

Y Species are provided for <u>all animals</u> within the study and motivated (*relevance* and *justification* is given).

I Species are provided to some extent, such as for some but not all animals, and may or may not be motivated.

N No mention of species.

<u>Unjustified duplication</u>

> SJVFS 2019:9 Ch. 2 Section 15 p. 8 *In what way unjustified duplication of animal experiments shall be avoided.*

Unjustified duplication

Y *How* the project has been planned to avoid unjustified duplication is described.

I *That* the project has been planned to avoid unjustified duplication is described but not how.

N No mention of avoidance of unjustified duplication.

<u>Housing and care</u>

> *SJVFS 2019:9 Ch. 2 Section 15 p. 11 An account of how the laboratory animals are to be kept and cared for before, during and, if relevant, after the animal experiment.*

Please note:

- How the animals are to be kept and cared for *before*, *during* and *after* the animal experiment is analysed and graded separately.
- The additional grades “Euth” or “Other” may be applied for *after* the animal experiment when the application clearly describes euthanasia/rehoming/reuse/release or return to owner as the end of the study for all animals. Meaning that there will be no animals left after the experiment to be housed and cared for by the researcher.

Housing and care

before the animal experiment

Y — *How* the animals are to be kept and cared for *before* the animal experiment is described for <u>all animals and procedures</u>.
This may be done separately for all animals/animal groups and/or procedures, or the standard housing conditions of the research facility may be described, so long as it is clear that they apply to all animals and/or aspects of the study.

I — *How* the animals are to be kept and cared for *before* the animal experiment is described to some extent.
It may be is described for some but not all animals/animal groups and/or procedures, or the standard housing conditions of the research facility are described but it is unclear if these apply to all animals and/or aspects of the study.

N — No description of how the animals are to be kept and cared for *before* the animal experiment.

during the animal experiment

Y — *How* the animals are to be kept and cared for *during* the animal experiment is described for <u>all animals and procedures</u>.
This may be done separately for all animals/animal groups and/or procedures, or the standard housing conditions of the research facility may be described, so long as it is clear that they apply to all animals and/or aspects of the study.

I — *How* the animals are to be kept and cared for *during* the animal experiment is described to some extent.
It may be is described for some but not all animals/animal groups and/or procedures, or the standard housing conditions of the research facility are described but it is unclear if these apply to all animals and/or aspects of the study.

N — No description of how the animals are to be kept and cared for *during* the animal experiment.

after the animal experiment

Y — *How* the animals are to be kept and cared for *after* the animal experiment is described for <u>all animals and procedures</u>.
This may be done separately for all animals/animal groups and/or procedures, or the standard housing conditions of the research facility may be described, so long as it is clear that they apply to all animals and/or aspects of the study.

I *How* the animals are to be kept and cared for *after* the animal experiment is described to some extent.
It may be is described for some but not all animals/animal groups and/or procedures, or the standard housing conditions of the research facility are described but it is unclear if these apply to all animals and/or aspects of the study.

N No description of how the animals are to be kept and cared for *after* the animal experiment.

Euth Euthanasia.
The application clearly describes euthanasia as the end of the study for all animals. There is as such no *after* other than death.

Other Rehoming, reuse, release or return to owner.
The application clearly describes one or several of the above as the end of the study for all animals. There is as such no *after* to be performed by the researcher.

End-points and euthanasia

SJVFS 2019:9 Ch. 2 Section 15 p. 13 Use of mild humane end-points and end-points as well as planned killing methods for the animal experiment.

Scientific end-point(s)

Y *Scientific* end-point(s) is/are described for the study.

N No mention of *scientific* end-point(s). Humane end-point(s) may be inaccurately described instead.

Humane end-point(s)

Y *Humane* end-point(s) is/are described for all procedures.
These are easy to understand, contain clear assessment criteria and are relevant for the animals used and the specific procedures planned.
If clear assessment criteria are not described by the applicant within the allocated space, an assessment template for evaluating the animals health, behaviour, pain or other indicators of reduced welfare (for example from Karolinska Institutet or Uppsala University) is specified for use and provided as an appendix to the application.

I — *Humane* end-point(s) is/are described to some extent and may or may not: lack clear assessment criteria; be relevant for the specific species or procedure(s) carried out; or apply to all species.
Generic phrases such as *"complications"* or *"manifestation of side effects"* may be used. An assessment template may be referenced but not provided as an appendix.

N — No mention of *humane* end-point(s). Scientific end-point(s) may be inaccurately described instead.

Methods of euthanasia

Y — Methods of euthanasia are described for <u>all animals</u> within the study (with the exception of individuals destined for rehoming, reuse, release or return to owner).

I — Methods of euthanasia are described to some extent, such as for some but not all animals to be euthanised.

N — No descriptions of methods of euthanasia for any of the animals within the study even though it is clear that animals will be euthanized at end of study.

N/A — Not applicable.
No animals will be euthanized at the end of the study.

Harm-benefit analysis by applicant

No regulatory requirement.<br>*According to application form: "Own ethical balancing".*<br>*The applicant's own description of how they have reasoned when reaching the conclusion that the benefit of the experiment exceeds the suffering of the animals.*

Please note:

- Harm and benefit are analysed and graded separately.
- There is no legal requirement that the applicant should perform a HBA when planning a project. It is however requested by the Swedish application form and hence included for evaluation in our study.
- This section is analysed and graded separately from the aforementioned legal requirements where the applicant is asked to describe the project's purpose, benefit and proposed severity classification (SJVFS 2019:9 Chapter 2 Section 15 p. 4).
- Directive 2010/63/EU and the L150 only specify that the harm and benefit should *balance out*. However, the application form asks that the applicant motivate why they believe the benefit *exceeds* the harm. Due to this discrepancy, both ways of reasoning by the applicant are considered acceptable in the study (project) when analysing the information below.

Harm

Y	The harm/suffering caused throughout this particular project is described with regards to *intensity/frequency and duration.*
I	The harm/suffering caused throughout this particular project is described to some extent and *intensity/frequency and duration* may or may not be given. Generic phrases such as *"we believe that the benefit of the project surmounts the suffering of the animals"* may be used.
N	No mention of the harm/suffering caused throughout this particular project.

Benefit

Y!	The expected benefit of this particular project is described.<br>*That* there is expected benefit is not enough, rather *what* and/or *how great* said benefit is expected to be must be specified.<br>The *likelihood* of achieving said benefit(s) is also mentioned.
Y	The expected benefit of this particular project is described.<br>*That* there is expected benefit is not enough, rather *what* and/or *how great* said benefit is expected to be must be specified.<br>The *likelihood* of achieving said benefit(s) is not mentioned.
y	*That* there is expected benefit of this particular project is mentioned but not *what/how great* said benefit is expected to be.<br>The *likelihood* of achieving benefit(s) may or may not be mentioned.
I	The expected benefit of this particular project is not described but benefit(s) of similar research or of an enveloping broader research area is.<br>Generic phrases such as *"increased knowledge about the underlying causes of diseases is of great importance for the development of efficient drugs*" or "*the need for treatment is grea*t" may be used.
N	No mention of benefit.

Information within the Non-Technical Project Summary (NTS):

SJVFS 2019:9 Ch. 2 Section 16 The application for ethical approval of animal testing shall be accompanied by a non-technical summary. The non-technical summary shall…

Please note:

- The contents of the "main technical body" of the application form need to be known in order for the non-technical project summary (NTPS) to be analysed in a correct manner. The language may be simpler in the NTS as it may be used by laypersons (and the public) as basis for their understanding of the project, but important information should for the same reason not be left out.

Purpose and benefit

SJVFS 2019:9 Ch. 2 Section 16 p. 1 inform about the purpose and benefit of the animal experiment,

Please note:

- Purpose and benefit are analysed and graded separately.

Purpose

Y The purpose of this particular project is described.

I The purpose of this particular project is not described.
The purpose of similar research or of research within the same field might however be.
Generic phrases such as *"the study of immunological disorders in mice aims to give insight into the hereditary aspects of similar diseases in humans"* or *"a previous study aimed to…"* may be used.

N No mention of purpose.

Benefit

Y! The expected benefit of this particular project is described in a manner and language which laymen may be expected to understand.
That there is expected benefit is not enough, rather *what* and/or *how great* said benefit is expected to be must be specified.
The *likelihood* of achieving said benefit(s) is also mentioned.

Y The expected benefit of this particular project is described in a manner and language which laymen may be expected to understand.
That there is expected benefit is not enough, rather *what* and/or *how great* said benefit is expected to be must be specified.
The *likelihood* of achieving said benefit(s) is not mentioned.

y *That* there is expected benefit of this particular project is mentioned in a manner and language which laymen may be expected to understand, but not *what/how great* said benefit is expected to be.
The *likelihood* of achieving benefit(s) may or may not be mentioned.

I The expected benefit of this particular project is not mentioned in a manner and language which laymen may be expected to understand, but benefit(s) of similar research or of an enveloping broader research area is.
Generic phrases such as *"increased knowledge about the underlying causes of diseases is of great importance for the development of efficient drugs"* or *"the need for treatment is great"* may be used.

N No mention of benefit.

Suffering

SJVFS 2019:9 Ch. 2 Section 16 p. 2 inform about the suffering of the laboratory animals,

Please note:

- If for example specific procedures are described within the provided space of the application but the negative effects/ suffering associated with them is not, the applicant still has not fulfilled the requirements for Y or I for this particular criterion and may only be graded as N.

Suffering

Y The harm/suffering caused throughout this particular project is described thoroughly with regards to *intensity/frequency and duration* in a manner and language which laymen may be expected to understand.

I The harm/suffering caused throughout this particular project is described to some extent and *intensity/frequency and duration* may or may not be given. Generic phrases such as *"symptoms will only arise at the end of the study"* or *"the suffering is not expected to be significant"* may be used.

N No mention of the harm/suffering caused throughout this particular project.

Number and type of animals used

> *SJVFS 2019:9 Ch. 2 Section 16 p. 3 include information on the number and type of laboratory animals to be used,*

Please note:

- Number and type(s) of animals used are analysed and graded separately.

Number

Y Number of animals used is provided.

N No mention of number of animals used.

Type

Y Type(s) of animals used is/are provided.

N No mention of type(s) of animals used.

The 3Rs within the non-technical project summary

> *SJVFS 2019:9 Ch. 2 Section 16 p. 4 show how the requirements for the 3R-principle are fulfilled according to Ch. 7 Section 1 of the Animal Welfare Act (2018:1192).*
>
> *SFS 2018:1192 Ch. 7 Section 1 Animal experiments may only be performed provided that: 1. the purpose of the activity cannot be attained by any other satisfactory method that does not use animals; 2. as few animals as possible are used; 3. the activity is designed in such a way that the animals are not subjected to greater suffering than is absolutely necessary; and 4. no animals other than animals bred for the purpose are used in the activity (purpose breeding).*

Please note:

- Legal requirements regarding 3R for the applicants includes an emphasis on *how* the requirements have been met, not just *that* they have been met.
- Only the R addressed is eligible to be graded. If one R has been inaccurately described as another, the R being described by the applicant is to be graded as Y, or I. It should however be specified in the Excel sheet that said description was ascribed the wrong R.
- The grading of the 3Rs in this section applies to whatever is specified about them within the realms of the NTS!

Replace

Y *How* Replacement has been considered for this particular project is described in a manner and language which laymen may be expected to understand.

Why animals need to be used and/or a certain animal model has been applied in this case is described, not just that "animal models are the only available options" or "the proposed model is standard" etc.

I — *That* Replacement has been considered for <u>this particular project</u>, but not how, is described in a manner and language which laymen may be expected to understand.
Generic phrases such as *"the proposed model is the most established model"*, *"no alternative methods exist"* or *"for the proposed study, live animals are needed"* may be used.

N — No strategy for consideration or application of Replace is mentioned.

Reduce

Y — *How* Reduction has been applied to and fulfilled within <u>this particular project</u> is described in a manner and language which laymen may be expected to understand.
How the applicant has ensured the use of the least possible number of animals is described, not just that "we have used as few animals as possible" etc.

I — *That* Reduction has been applied to and fulfilled within <u>this particular project</u>, but not *how*, is described in a manner and language which laymen are expected to understand.
Generic phrases such as *"by carefully planning the project, the number of animals needed has been kept to a minimum"* or *"only as many animals as are required to obtain scientifically valid results will be used"* may be used.

N — No strategy for consideration or application of Reduction is mentioned.

Refine

Y — *How* Refinement has been applied and fulfilled within <u>this particular project</u> is described for <u>all</u> procedures in a manner and language which laymen are expected to understand.

I — *That* and/or how Refinement has been applied within <u>this particular project</u> is described to some extent in a manner and language which laymen are expected to understand.
Generic phrases such as *"only educated staff handles the animals"* or *"the methods used are well-known and reliable"* may be used.

N — No strategy for consideration or application of Refinement is mentioned.
